# The Hospital Nursing Supplement

**Published:** 1896-10-17

**Authors:** 


"The Hospital" Nursing Mirror.

Being the Extra Nursing Supplement of "The Hospital" Newspaper.

[Contributions for this Supplement should be addressed to the Editor, The Hospital, 28 & 29, Southampton Street, Strand, London, W.C., and should have the word "Nursing" plainly written in left-hand top corner of the envelope.]

### News from the Nursing World.

### THE EARL OF MEATH ON IRISH WORKHOUSE NURSING.

At a recent meeting of the Rathdrum Board of Guardians a proposal to appoint a qualified night nurse was seconded by the Earl of Meath, who took the opportunity to strongly condemn the barbarous custom which largely prevails in Irish workhouse sick wards of locking the unfortunate patients up without any nurse from five in the evening to six the next morning. He pointed out that "during the few hours of day the patients in the infirmary had a nurse, while for the larger number of hours and at a time when the temperature of the patients was lowest and death most frequent they were without any attendance. In his opinion it was simply cruelty." We rejoice to see that it was finally unanimously decided to appoint a properly trained night nurse at a salary of £25 per annum.

### AT THE WOMEN'S CONGRESS.

The ladies—literary, scientific, medical, and socialistic—who have been taking part in the International Congress of Women at Berlin have dispersed to their various parts of the world well satisfied with the success of their meeting. Several Englishwomen took part in the discussions; amongst others, Miss Georgiana Hill read a good paper on "Women as Poor Law Guardians." The paper on "Nursing" contributed by Fräulein Anna Stosch, herself the proud possessor of two Crosses of Honour awarded for her work during the Franco-German war, dealt particularly with the Victoria Home for Nurses, established fourteen years ago in Berlin by the Empress Frederick. At the present time the home contains some two hundred and fifty sisters, nurses, and probationers. After hearing this account of the home the visitors went to see it for themselves, and were favourably impressed by the conditions under which these German nurses carry on their work.

### INVALID COOKERY CLASSES.

Demonstrations on the preparation of sick-room food are to be given this winter at the offices of the Royal British Nurses' Association (17, Old Cavendish Street), beginning next month on Tuesday afternoons at half-past two. Many nurses who are not fortunate enough to have bad instruction in cookery included in their hospital training will be glad to avail themselves of this opportunity. The tickets for the full course (which are for members 7s. 6d., for non-members 12s. 6d.), will be transferable for the convenience of those who may like to share the expense with a friend. Each demonstration will last for two hours. The names of those wishing for tickets should be sent in to the Secretary before October 24th.

### NURSING HOMES AS PRIVATE VENTURES.

We have always set our face against the sweating of nurses. It is notorious that large sums of money have been made in the past, and no doubt are being made in the present, by those who conduct private nursing homes for their own profit. Homes so conducted must necessarily pay the nurses much less than they earn, and are consequently undesirable from the point of view of the public, as well as from that of the nurse. The recent split in a northern town, which is creating a good deal of animosity on both sides at the present time, shows that these drawbacks, great as they are, do not exhaust the evils attaching to the private venture home. The leading citizens of our great towns would do a considerable service to the nursing profession if they were to form a committee and organise a nurses' co-operation for the supply of private nurses to the public on the lines adopted by the Nurses' Co-operation, 8, New Cavendish Street, London, W. They must, however, take care to see that no one is permitted to obtain a seat on the committee who does not possess sufficient capacity and intelligence to take a clear and businesslike view of the matters which may come before them for settlement.

### A PAY HOSPITAL IN PARIS.

The patients at Maison Dubois, a paying hospital under the control of the Paris Municipality, managed by the *Assistance Publique*, seem to be in an unenviable position. The head surgeon, Dr. Picqué, has been complaining for months past of the insufficiency of the nursing, and matters reached a crisis lately when, after the performance of two serious operations, there was no nurse available to attend to the patients. Dr. Picqué now declines to perform any further operations at the hospital until some reform has been effected, and points out that the administration undertakes by contract to place a nurse at the disposal of each patient admitted, for whom the patient pays, and, failing to carry out this engagement, the hospital should be closed. The nurses sent by the *Assistance Publique* in response to the doctor's representations are declared by him to be "ignorant, dirty, or even worse."

### INFIRMARY NURSES AND THE NEW SUPERANNUATION ACT.

Our advice has been asked by several nurses who at present belong to the poor law service, or who contemplate entering that service, as to what they should do in view of the passing of this Act. Under this Act every infirmary nurse who is at present in the service, unless she contracts herself out of the Act, will forfeit all her contributions to this fund unless she remains in the service until she is sixty or sixty-five years of age. Any nurse not at present in the service who may now join it will have to accept the Act as a condition of her election, and must therefore submit to the forfeiture of the whole of her compulsory contributions under it should she voluntarily resign. But if she is "dismissed for any offence of a fraudulent character, or for grave misconduct," the Guardians may, if they see fit, return to her all her contributions under the Act. We have always had a very poor opinion of poor law administration under Guardians, but Parliament has quite outstepped any such moderate estimate by the irony contained in Clause 4, entitled "Forfeiture for Fraud." In the face of an Act drawn without any regard whatever to the claims and rights of nurses, passed, as we understand, despite an undertaking previously given to the nurses' representatives that those rights should be protected, we feel it a duty to warn all nurses at present outside the poor law service to remain outside until justice is done to the nurses in this matter. Our advice to the nurses who are at present engaged in the poor law service is to contract themselves out of the Act without a moment's delay, so as to protect themselves from a pecuniary loss which is so unjust as to make it abominable. If and when those responsible for the injustices in question move Parliament successfully to secure the rights of nurses, then, but not till then, intelligent women who are trained nurses may be justified in joining the poor law service.

### THE ARCHBISHOP OF CANTERBURY AT ST. PATRICK'S HOME, DUBLIN.

The sorrow caused throughout the country by the news of the tragically sudden death of the Archbishop of Canterbury at Harwarden has been deep and genuine. Archbishop Benson had but just returned from Ireland, from a visit of which he is said to have spoken "with great delight," though it was by no means a mere holiday. An account of a visit of the Primate to St. Patrick's Home, Dublin, the headquarters of the Queen's Jubilee Institute for Nurses in Ireland, has come to us from an English nurse working there, written before the sad news was known. The Archbishop had expressed a wish to see the nursing institutions attached to each cathedral, so, after preaching at the choral evensong at St. Patrick's Cathedral on the afternoon of Sunday, September 20th, he accompanied Lord Plunket, Archbishop of Dublin, who is chairman of the St. Patrick's Home Committee, to the Home, where a number of guests had been invited to meet him, amongst others, the Bishop of Glasgow. The Archbishop talked pleasantly to the nurses, who one and all "thought it extremely kind of him to come and see them," and he was shown over the Home by Miss Howell, the superintendent, leaving, when he went away, "very pleasant recollections in the minds of the nurses."

### QUEEN VICTORIA'S JUBILEE INSTITUTE.

The gold badge of Queen Victoria's Jubilee Institute has, with the approval of Her Majesty the Queen, been given by the Council to Miss Pauline Peter, inspector of the Institute, and to Miss Mary Dunn, superintendent of its branch in Ireland. The silver badge has been given by the Council to Miss Agnes Wells, superintendent of the Haggerston and Hoxton District Nursing Association; Miss Elizabeth A. Walker, superintendent of the Bolton D.N.A.; Miss Sara Wilson, superintendent of the Shaw Street Division, Liverpool, D.N.A.; Miss Jessie Blower, superintendent of the Ardwick Green Division, Manchester, D.N.A.; and Miss Jane Brown, superintendent of the Leeds D.NA. On the recommendation of the Council of the Scottish Branch, the following superintendents in Scotland have also received the silver badge from the Council of the Institute: Miss Isabella Armstrong, superintendent of the Aberdeen D.N.A.; Miss Isa Watson, superintendent of the Paisley D.N.A.; Miss Mary Berwick, district superintendent of the Glasgow S.P. and P.N.A.; and Miss Haddon, assistant superintendent of the District Training Home, Edinburgh.

### BAZAAR AT ST. HELENS.

Lady Gerard, who interested herself greatly in a bazaar lately held in aid of St. Helens' "Providence" or Free Hospital, gave a reception at the hospital the other day to all those who had worked with her to make it successful, and nearly three hundred guests accepted her invitation. Their efforts were quite phenomenally satisfactory, for the bazaar resulted in a sum of nearly £1,300 towards the £1,500 required to clear off debts and carry out improvements. The nursing at this hospital is in the hands of Roman Catholic sisters.

### DISTRICT NURSING ASSOCIATION FOR BRIGHTON.

In consequence of the old "Sick Poor Nursing Association" at Brighton having ceased to exist an entirely new association has been started, which includes Hove and Preston in its area. The headquarters of the new organisation, called the "Brighton, Hove, and Preston Association for Nursing the Sick Poor," and affiliated to the Queen Victoria Jubilee Institute, are at 5, Marlborough Place. The home was ready for occupation on October 1st, when the staff were duly installed, and the nurses have already begun their work. A public opening and meeting on behalf of the society will take place next month. The Bishop of Chichester has accepted the presidentship, the Duke and Duchess of Fife and the Earl and Countess of Chichester are patrons, and the Vicar of Brighton, the Rev. Prebendary Hannah, is chairman of the Executive Committee. Miss Buckle has been appointed lady superintendent.

### THE ZENANA MISSION.

The usual farewell meeting to missionaries departing for India under the auspices of this society took place on October 16th. Three of the nine ladies going out this autumn are returning after leave of absence. Of these, Miss Townsend goes to the Lady Kinnaird Memorial Hospital, Lucknow; and, of those taking up work among the women of India for the first time, Miss Riley, who is a trained nurse, goes with Miss Owston to the Duchess of Teck Hospital at Patna. Sixteen large packing-cases, the results of many working parties among friends at home, accompany the missionaries. Lately the society has received the sum of £10 for the support of the "County Wexford Cot" in the Lucknow Hospital, while a similar amount has come through a friend in Liverpool for a bed at the Benares Hospital.

### A "REPRESENTATIVE ORGAN" AND ITS NURSING NEWS.

It is interesting to note how a contemporary which describes itself as the "representative organ of the nursing profession" obtains its news. In the *Daily Telegraph* for October 5th appeared, without acknowledgment, a statement taken from The Hospital of October 3rd, as to the organization of a nursing home in Shanghai. In that statement was a material error, Nurse Gladwell's name being given as "King." Last week the "representative organ" took this paragraph from the *Daily Telegraph*, without acknowledgment, and carefully included this mistake, which we now have much pleasure in correcting.

### NEW NURSES' HOME AT THE ROYAL HOSPITAL FOR SICK CHILDREN, GLASGOW.

Extensions are in contemplation at the Royal Hospital for Sick Children, Glasgow. The present nurses' quarters are to be given up for the enlargement of the dispensary, while a new building is to be erected to the west of the hospital for the accommodation of the nursing staff. The estimated cost of these improvements is about £1,700.

### Hygiene: For Nurses.

By John Glaister, M.D., F.F.P.S.G., D.P.H.Camb., Professor of Forensic Medicine and Public Health, St. Mungo's College, Glasgow, &c.

### XXVIII.—CLOTHING IN RELATION TO HEALTH.—MATERIALS EMPLOYED IN MANUFACTURE.—THEIR USES AND VALUES.

The primary function of clothing, of whatever kind, is to conserve the animal heat of the body, and it combines with this duty the subsidiary purposes of serving for decency, for the adornment of the person, and for protection against injury. Conservation of the body-heat is especially necessary in temperate and arctic climates. In warm or equatorial climates, on the other hand, it fulfils the purposes of personal adornment and bodily protection chiefly. The study of clothing has received of late years closer attention than ever it did before, especially from the hygienic standpoint. The human body is capable of resisting heat and cold through a very considerable range of temperature, but it is more able to withstand extremes of heat than of cold. This might be predicated from the fact that the animal body is a great heat producer in relation to its size, food being the fuel, and the bodily organs the furnace in which the fuel is burned by chemical action; but the total amount of heat generated within a given period is, however, limited at the best. The object of clothing, therefore, is to prevent this body-heat being dissipated too rapidly from the body. The materials of which the different fabrics are composed are obtained almost equally from the animal and vegetable worlds.

From the former are obtained (1) wool, mainly from the sheep species, but partly from the goat species, and from the camel, alpaca, and vicuna; (2) silk from the cocoon of the silk-moth, and from other silk-yielding moths; (3) skins, which are used after preparation, with or without adherent fur; (4) hair; and (5) feathers of birds; from the latter (1) cotton, from cotton-shrubs; (2) linen, from the flax plant; (3) jute and hemp, used for subsidiary clothing purposes; (4) guttapercha, or caoutchouc, from the indiarubber plant; and (5) straw, from wheat stalks and grasses, cut when green, and afterwards bleached.

These different stuffs are used either pure or mixed, and compose, in the main, the different fabrics of which body-coverings are made. A rapid review of these coverings will show how these materials are utilised for body protection and heat conservation. For example, in the head-gear of both sexes, straw, silk, satin, feathers, fur, wire, linen as artificial flowers, cotton, wool, felt, and like materials, find a place. Linen composes the collar and collarette, and it also, like cotton, wool, and silk, enters largely into the composition of underclothing. The outer person is clad in garments of wool, silk, or cotton. Leather is found in boots, shoes, vests, and other articles of attire, while guttapercha enters into the composition of foot-gear and overalls, for protection against damp. Furs serve the double purpose of adornment and heat conservation, chiefly for the former in temperate climates, and solely for the latter purpose in arctic climates. The materials of which textile fabrics are made differ both as to their physical and microscopic appearances. A good hand magnifying glass will reveal these differences. Wool fibres appear as continuous stems, with circular markings, which give the edges of the fibre a notched character, owing to which they more readily lend themselves to the operations of twisting, weaving, and matting. Silk-fibres look like clear threads, without any pith; cotton-fibres have roughened edges, and show twistings in their length; linen-fibres exhibit nodular points in their length; and it is difficult to distinguish between jute and hemp. Their different behaviour when treated with certain chemicals and dye stuffs further helps to distinguish between them.

Permeability in Respect of Air.—Different cloth stuffs, of exactly similar size, when subjected to the same test, exhibit differences in the respective amounts of air which may be passed through them in a given time. Suppose flannel, linen, silk, and leather to be taken for the test. The result will show that 100 parts of air will pass through the flannel for 60 of the linen, 40 of the silk, and about 30 of the leather. The porosity or permeability of these substances will, therefore, be in the order named.

Water-Absorptive Capacity.—If pieces of new silk, linen, cotton, and flannel of exactly similar size be taken and placed flat-ways and lightly upon the surface of cold water, they do not float for equal periods of time. Silk sinks almost at once, linen in about one minute, cotton in about four and a quarter hours, and flannel does not sink at all. This experiment shows, approximately, the rate at which garments made of these respective stuffs will become saturated with damp. If worn materials be used in the experiment, and warm instead of cold water, absorption is relatively more rapid.

Thermic Properties.—The heat-conducting capacity of different textile fabrics is by no means the same. Cotton and linen, especially the latter, feel colder to the touch than silken or woollen articles. They are, therefore, better heat conductors than the latter; that is to say, in the same time they rob the body of more heat. This conductivity does not, however, depend so much upon the materials themselves as upon the closeness or openness of the woven texture. The "flufflier" the material the more air is held within the interstices of the fabrics.

The roughness of the fibre of wool as compared with those of linen, or cotton, or silk, by setting up some degree of irritation of the skin, and, therefore, a greater supply of blood to that part than usual, doubtless contributes in some measure to the greater sense of warmth of a woollen garment, and, for a like reason, is ill-borne by hyper-sensitive persons.

Behaviour to Sensible Perspiration.—This is a matter of considerable practical importance. Cotton, linen, and wool behave differently. Experience amply demonstrates that, when garments worn next the body, and composed of these materials respectively, become wet with sweat, a feeling of chilliness is more quickly perceived in the case of cotton and linen than of wool. Heat, therefore, is more rapidly abstracted from the body in the case of the two former than of the last. Hence chills are more liable to happen when cotton or linen is so worn. It may safely be reckoned that, in the same time, damp cotton or linen abstracts not less than 30 per cent. more heat from the body than does damp wool. They also act differently in regard to the solid and liquid portions of sweat. Cotton and linen retain more of the solids than flannel or wool, and, therefore, more quickly become apparently soiled than wool, whereas wool permits the "solids" to pass through, to soil the garment worn next above, while it itself remains apparently longer clean.

Colour and its Relation to Heat.—The colour of a garment has an important bearing on the amounts of radiant heat absorbed and reflected. White and the lighter coloured fabrics absorb less heat than dark coloured or black, and reflect more; and the differences in amounts are graded by the tones of colour between white and black.

The dust-carrying capacity of clothing, and, therefore, facility for conveyance of infective material, depends in a large degree on the roughness or smoothness, and the openness or closeness, of texture of the fabric. The rougher and more porous the garment the greater the capacity for carriage of dust or contagia; and the converse is equally true. Therefore, the dresses of nurses, while tending the sick, ought to consist of close-textured material, capable of being frequently washed and ironed, so that a glazed smooth surface may be formed, and thus a less suitable resting-place for microbes be offered.

The colour of a garment has no relation to the odour-absorbing capacity of the fabric, as has been asserted. This has probably arisen from the fact that the average colour of the dress in cities is of a dark shade, and is usually associated with woollen fabrics, which, because of their porosity, absorb more odour than close-textured stuffs.

What should be worn to preserve health? is a most important consideration, as no one dress standard is equally suitable for all points of the globe. The discussion of the question will be simplified if we deal with it in reference to typical climatic conditions. There are four such typical kinds, the intervening grades being but modifications of one or other type, viz.: (1) the cold or arctic climate; (2) the hot, dry climate; (3) the hot, moist climate; and (4) the so-called temperate climate. These are but arbitrary divisions, and must not be confounded with the meteorological definitions of climate. In the first-named, the greatest struggle to maintain the body-heat is experienced. This is not difficult to understand when it is remembered that such temperatures as 69 deg. Fahr. and 81 deg. Fahr. below freezing point have been recorded, and where, even in July, the thermometer hardly rises above freezing-point. Experience has shown that the cold can only be overcome by wearing woollen garments next the body, and over these the skins of animals with the furry side inwards. No other forms of clothing will adequately conserve the body-heat. In the second the nature of the clothing is entirely different. The air being dry and greedy of watery vapour, sensible perspiration is usually absent. Comfort, therefore, will more likely ensue from the wearing of white, grey, or other light-coloured garments composed of cotton, linen, or wool, made loose-fitting; light-coloured the better to reflect the sun's radiant heat, and lined, preferably with a dark-coloured lining, to prevent the scorching effect of the solar heat rays, and loose-fitting, to enable free interchange of air. Sunstroke is avoided by the pith hat, puggaree, or sombrero, or Panama straw hat. Where the climate is hot, but moist—the third type—where the air is commonly saturated with watery vapour, and where sensible perspiration is almost never absent from the body, except when in absolute repose, woollen garments are absolutely indispensable both for night and day wear. Loose-fitting they may be, but wool they must be. In the fourth type—the so-called temperate climate—more difficulty is experienced in regulating the proper clothing than in any of the previous, solely due to the unexpected fluctuations of temperature. Thus, we may have summer temperatures in winter and winter temperatures in summer. In these islands there also the additional fact that the atmosphere is largely charged with watery vapour, and particularly on the west coast. These disadvantages, however, are compensated for by our proximity to the Gulf Stream, which alone prevents our winters from being of arctic severity. There is no season of our average year in which woollen underclothing can be dispensed with. It may be thicker or thinner in make, close or open in texture, as the weather permits, but it must be constantly worn, consistent with the safety of the person. Indeed, nothing can be said too strong in urging this upon our populations, as attention to it would considerably minimise illness and temper its severity.

### Trained Nurses' Clinic.

### XIII.—THE NURSING OF CHILDREN (*continued*).

The wise personal influence of a skilled nurse must not be confounded for a moment with that power which is sometimes acquired over children by unprincipled people through the medium of fear. The first is exercised exclusively for the sick person's own benefit; the second is improperly acquired and improperly used. It is a source of such harm as can scarcely be over-estimated, for the fears of childhood are easily raised but hardly controlled. Anybody who obtains even temporary ascendancy over a sick person by means of fear may be unhesitatingly condemned as unfit to be entrusted with the charge of either healthy or sick children. The exercise of her influence can do nothing but harm both mentally and morally, and there should be no delay in removing such a woman from the sick-room or nursery.

Children are often the unsuspected victims of an amount of tyranny of which their natural guardians remain complacently ignorant. Few young children ever dream of appealing against harsh treatment which comes from what they believe to be an authorised source. Even the boldest children feel very weak and feeble when they find themselves opposed to the will and strength of an adult. The helplessness which forms their claim on the tenderness of their elders renders them easy victims to the harshness of such persons as lack knowledge or sympathy.

But the influence of the wise "children's nurse" can be exercised to the advantage of all with whom she comes in contact. It originates in her comprehension of the natures of children, allied to accumulated knowledge of the treatment they require, this knowledge being the outcome of long practical experience. Such a nurse does not regard children simply as miniature men and women from whom a large measure of reasonableness and patience can be rightfully demanded. She asks but little from her young charges, but she gives lavishly of her best powers and care, and is generally repaid by the unstinted confidence which is yielded to her. Children accept the decisions of those in whom they believe with wonderful alacrity. They have unlimited faith, and exhibit it to a remarkable extent after their confidence is once gained.

The nurse who gives confidence in return, letting her patients see that she trusts them, and appreciates their little efforts to be brave and obedient, is well repaid by an increased display of both qualities.

Frequently sick children are considered by their relatives to be quite too ill to do what they are told, and their intermittent obedience to the doctor is only secured by continuous bribery or by even more foolish prognostications as to the evil results of neglecting his orders.

Such experiences as these add materially to the difficulties of the trained nurse, whose introduction to the child perhaps takes place when he is sufficiently ill to need constant attention, which he may resent at the hands of a stranger and refuse from his own relations.

Infinite tact, gentleness, and sympathy are demanded of the nurse who is suddenly imported at this critical time. In a ward the traditions of discipline, the influence of example, and the frequent visits of the medical officers tend to uphold and to strengthen her authority. Even a young patient is fully alive to the fact that the doctor can be fetched at any moment of the day or night; his appearance follows so promptly on the summons as to amaze the new patients.

All the influence in a hospital lies on the side of law and order, and the children are instinctively docile. Of course, there are exceptional cases, but the average boy or girl may be depended on to behave much better within the hospital than at home.

It is, therefore, in private households that the nurse finds most difficulty in enforcing the requisite amount of obedience.

Mothers and fathers, aunts and uncles do not hesitate to call the sick child troublesome, disobedient, unruly, &c., amongst themselves; but they all unite in representing him to the nurse as the incarnation of good. They resent any criticism not uttered by themselves, and in their fear lest nurse should be too strict, they often do their best to cripple her power, to undermine her authority, and to make her feel an outsider, and, therefore, the child's natural enemy.

(*To be continued.*)

### Nurses in 1896—Their Quarters, Hours, and Food.

[These articles exhibit the actual condition of affairs in the spring of the present year.]

### ST. MARY'S HOSPITAL.

### I.—TERMS OF TRAINING.

PROBATIONERS are admitted to St. Mary's Hospital for a term of three years' training, candidates being required to be between the ages of 25 and 35 years, of average height and physique, and to enter on a month's probation. The certificate of the hospital is given at the conclusion of the three years during which they are engaged to serve in its wards, and during this time they are expected to attend regular courses of lectures, given by various members of the medical staff, on medical, surgical, and obstetric nursing, and to pass examinations on the instruction given. A limited number of paying probationers are received at St. Mary's, for one year's instruction in nursing, at the end of which time they are entitled to a written statement to the effect that they have had a certain amount of training, such a statement not to be confounded with the "hospital certificate" accorded to those who have followed the three years' course of training. The fee for this one year is £30, or if a separate bedroom is desired (a nurse's necessity still, unfortunately, by force of circumstance deemed a luxury at St. Mary's) £50. The fees are payable in quarterly instalments in advance. The duties of paying probationers are the same in all respects as those of ordinary or paid probationers in their first year.

Nurses are not promoted to be sisters of wards until they have passed through the full term of three years' training. Paying probationers who wish to complete their training and qualify for the certificate may, if there be a vacancy and they are approved by the matron, be transferred to the regular staff.

### II.—HOURS OF WORK AND TIMES OFF DUTY.

Day nurses and probationers go on duty at seven a.m., the former leaving the wards at night at nine p.m., the latter half-an-hour earlier. During these hours probationers are off duty daily from ten a.m. to twelve, or from half-past two to half-past four, and on alternate Sundays from half-past nine a.m. to a quarter-past one p.m., and from three p.m. to ten p.m. Off-duty times for nurses are differently arranged. They are not off duty every day, but twice a week from five p.m. to ten p.m., once a week from seven p.m. to ten p.m., and on Sundays their hours off are the same as the probationers—from half-past nine a.m. to a quarter-past one p.m., and from three p.m. to ten p.m. alternate weeks. Sisters' daily hours on duty are from eight a.m. to ten p.m., with leave twice a week from five p.m. to half-past ten p.m., once a week from seven p.m. to half-past ten p.m., on Saturdays from two p.m. to four p.m., and on alternate Sundays from half-past nine a.m. to twelve noon, and from a quarter-past one p.m. to three p.m. Besides these hours, one whole day each month is allowed alike to sisters, nurses, and probationers. Night nurses' hours are from nine p.m. to half-past eight a.m. They are off duty daily from ten a.m. to twelve noon, and they have also one whole night and day each month. Yearly holidays are: For the sisters, one calendar month in the summer; day and night nurses, three weeks in the summer; and for probationers, ten days every six months and three weeks at the end of two years.

Nurses are allowed to sleep away from the hospital on the monthly holidays, a concession which, no doubt, adds much to the advantage of the day off, where, as so often happens with London nurses, they have friends within easy reach. It will be seen on examination of the hours quoted above that nurses practically cannot get out-of-doors before five in the afternoon except on Sundays and on their days off, and sisters the same with the addition of the Saturday leave from two o'clock. Also on three days in the week day nurses are on duty from seven a.m. to nine p.m., which, subtracting three half-hours for meals, means twelve and a half consecutive working hours. Sisters have two similarly hard days in the week, being on duty from eight a.m. to ten p.m., save for meal times. The nurses get a fairly long night's rest if they go to bed promptly on finishing their supper, apparently about seven and a half hours on an average for day nurses and rather less for those on night duty.

### III.—MEALS.

Day nurses breakfast at half-past six, they have a light lunch at half-past nine, dinner at half-past twelve, tea from four to five p.m., and supper from half-past eight to half-past nine. Night nurses "breakfast" at half-past eight p.m., and dine at nine a.m., having also another light meal, or "lunch," before going to bed. Sisters' meals are served in their own sitting-rooms, with the exception of the mid-day dinner, which is served in the nurses' dining-room at half-past one p.m. The only "stores" given out are to the night nurses, and these are sent to the special keeping-place reserved for them in the ward. The dietary consists of tea, coffee, or cocoa, bacon, eggs, &c., for breakfast; cocoa and bread and butter for lunch; dinner of two kinds of joints, vegetables, and puddings; and cold meat, soups, hashes, and cheese for supper. The night nurses have two meals in the ward during the night, for which cold meat, eggs, and bacon are provided.

### IV.—SALARIES AND UNIFORM.

"Probationers, Class I.," or "regular" probationers, as paid probationers are sometimes called, receive in salary for their first year £12, for the second £15, and for the third £18. Staff nurses are paid £20 to £25 per annum, and sisters £30 to £40. Class I. probationers are supplied with indoor uniform, consisting of three print dresses, three caps, and eight aprons in the year, and they and the staff nurses, to whom a similar allowance is made, are entitled to what is specified in the regulations as a reasonable amount of washing at the hospital's expense. The paying probationers are required to provide their own uniform and to bear the cost of their washing. Outdoor uniform is not compulsory.

St. Mary's Hospital is affiliated to the Royal National Pension Fund for Nurses, the committee paying half the nurses' premiums entitling them to pensions at the age of 55 years.

### V.—NURSES' QUARTERS.

If the people who are interested in St. Mary's Hospital would take the trouble to visit the quarters at present allotted to the nursing staff on the top floor of the building, they would surely be impelled to energetic attempts at once to raise the money needed for the completion of the new buildings, which are to include a proper home for the nurses. This is very much needed, for the present accommodation is inadequate and out of date. There are some single rooms, but for the most part the nurses are placed two in a room, divided by a screen; while the probationers share a room among three, divided by curtains. There is a pleasant little sitting-room, with a balcony, comfortably furnished, though space is limited. The nurses' dining-room is in the basement, a good-sized room and fairly comfortable, but more space is urgently required for the comfort of the staff. Sisters have bed and sitting rooms off their wards.

### A Book and its Story.

### THE DREAM CHARLOTTE.*

Miss Betham-Edwards gives us a well-drawn picture of Normandy at the time of the French Revolution. Her romance centres round a quiet little homestead, historically interesting as being the scene of Charlotte Corday's earliest years. Here she was nurtured by the good farmer's wife, whose daughter, Airelle, is the heroine of the volume, and whose enthusiastic admiration for her foster sister forms the principal theme throughout the pages.

With Charlotte, Airelle passed her youth at a Norman convent, and we are first introduced to her on the occasion of leaving the seclusion of the convent and returning to her home life at the farm.

"The eighteen-year-old girl, their convent-bred daughter, seemed a portrait of both her parents, Isaye Aubery and his wife Gillette. Here was the same largeness, the same amptitude, yet toned down by spells more potent than youth or benignity. Isage and Gillette had given their heir an admirable physique, glorious beauty, and fine qualities of heart and mind. Another had imparted soul, outside influence and agencies subdued flesh to spirit. Child of these simple folks, she belonged to herself and her ideals."

And this "other" to whom Miss Edwards refers was no less august a personage than Charlotte Corday, who, even in her convent days, showed signs of marked personality.

The advanced young Revolutionary had her own political views at an early age, with which views Airelle became indoctrinated, and she is brimming over with somewhat advanced ideas on her return to the bosom of her family. Airelle's mother, whose domestic soul had not been stirred by the rumoured rising throughout the country, regarded such aspersions as almost criminal when her daughter broached the subject to her.

"But Charlotte understood them, the subjects I speak of," Airelle would argue. "We used to get news in the convent, of course; we heard of what was going on outside—the insurrection, the razing of the Bastille, the clamour for juster laws. And Charlotte grew wild with joy. She said that France was freeing herself from her fetters, that the reign of tyranny was over, and fanaticism doomed. Ask your own heart, mother; can it be right that one Frenchman helps to fill the King's treasury, fights the King's wars, be liable to torture and death without trial; whilst another, the noble, the privileged, possesses his purse, his freedom, the inviolability of his person? Can it be right that our old Judith's kith and kin were imprisoned, ruined, exiled simply for refusing to hear mass?"

It was "God's will," the mother argued, not wishing to enter into any argument wherein the validity of her religion was questioned.

"Look at yourself," the daughter continued. "You, although, perhaps, you hardly recognise it, condemned fanaticism by taking a so-called heretic into your service. Father Patrix disapproved of having Judith at the beginning."

The drawing of servant Judith's character is a strong feature in Miss Edwards' book. The serving woman is brought before us with much vividness, and forms a picturesque and pathetic study of an old Huguenot woman. It never occurred to Judith to arraign human injustice. Her poverty, her isolation, her sorrows she attributed to law, in her mind "a power akin to merciless hidden forces. That her husband had died for his faith, that her child had been kidnapped by Royal command, her kinsfolk outlawed, and home wrecked, were misfortunes ordained of Heaven, inevitable outcome of social order. Protestants were tortured, banished, put to death, because the law would have it so. But behind the law, backing up man's sins against his brethren, were agencies she could not explain, mysteries unreadable as the tragedy of Nature."

These were stirring times in France, and whilst something stronger than the law was making itself felt throughout the length and breadth of the land, this sketch of the paralysing effect of the down-treading of the lower classes is cleverly given by Miss Edwards. This universal injustice appears to have engendered a dogged acceptance of all social evils by the French peasantry at that period. The kindling voices which pressed on the nation for a new state of things did not come in any one case from the peasant, but from the outsider and onlooker. This little volume clearly shows us how the daily round, the common task engrossed in an all-sufficient manner the thoughts and aspirations of the agricultural classes, crushing out for the time being all spirit of resistance. Their world was principally confined to their tillage grounds: there was no real public press, and no outside postal communication to facilitate a larger view.

Among the many who, at this tragic juncture in the history of France, rose up to redress her country's wrongs was Charlotte Corday—Airelle's "Dream Charlotte," as she terms her. Her story, an old familiar one to us, is too scantily dwelt on in Miss Betham-Edwards' book; it is mainly through the words and thoughts of her foster sister that we are brought in contact with her; only once or twice through the book she speaks for herself.

The last time the two girls meet, both filled with a patriotic enthusiasm, Charlotte Corday informs her friend she is about to undertake a daring experiment. She herself will confront Marat, "The Tyrant," as she terms him, and speak to him in the name of the "people." Airelle, ever ready to see the heroic in Charlotte's conduct, feels the strength of the young girl's purpose, and regards it in the light of an inspiration. "Not as yourself, my own foster sister, a simple girl of Normandy, will you appear before him, but as an advocate of France, our crushed, bleeding, gasping country. Your youth, innocence, and daring, will appal him; as in the presence of an avenging angel his black soul will take fright."

The two part, never to meet again. Charlotte Corday's mission was, as we know, something other than a mere message.

Some three days later, as Airelle drove up to the post-house near Lieon, she saw that the diligence had just arrived, bringing the latest news-letter from the Capital. "Some unusual excitement had emptied every farmhouse, cabin, and cabaret—every workshop of master and apprentice." A thought flashed across Airelle's mind—was there news of Charlotte, of her sublime mission, the era of proscription and bloodshed brought to a close—France saved by a woman's pleading?

Hats were thrown up, handkerchiefs waved, frantic huzzas filled the air; far and near folks shouted, "The Tyrant has fallen! the Tyrant has fallen! The Virgin and Saints be praised! France has her Jaels, her Judiths!"

Then it was forced upon the girl's bewildered senses—Marat was dead. "For a moment Airelle stood stone still, not a cry, not a feature betraying the passion of despair. No angelic messenger, then, had been her Charlotte; no heaven-sent Prophetess of just retribution. No softening of the tyrant's heart had prompted that strange journey."

Her dream Charlotte had vanished; in the room of a fondly-cherished memory must remain horror and shrinking; the Charlotte of proudest memories was replaced by a figure as beauteous to look at, but hemmed round with awful circumstances; she thought to save France, and to avert further bloodshed. But Airelle's loyalty was unshaken. The girl stoutly declared that "Her crime was prompted by love of country and fellow countrymen."

* "The Dream Charlotte." M. Betham-Edwards. (London: Adam and Charles Black. 1896.)

### City of London Union Infirmary.

GOVERNMENT CENSURE ON THE MEDICAL OFFICER AND MATRON.

At a meeting of the Guardians of the City of London Union on the 13th inst. a communication was read from Mr. Knollys, the Assistant-Secretary of the Local Government Board, embodying its decision in regard to the report of their inspector (Dr. Downes) of the official inquiry held by him into the administration of the Infirmary of the City of London Union, so far as relates to the differences which have arisen between the medical officer and the matron, together with the depositions of the witnesses examined at the inquiry. The Board have carefully considered the report and the evidence, and express their grave dissatisfaction at the disorganisation, for which they regard the medical officer as primarily responsible by reason of his general want of systematic procedure in regard to the administration of the establishment, and particularly in regard to the lack of discretion, deliberation, and judgment evinced by him in his intervention in the routine duties of the matron's department. The Board further express their disapproval of the hasty and inaccurate manner in which many of the statements and charges had been framed by the medica officer. As regards the conduct of the matron, the Board have, as also in the case of the medical officer, made full allowance for the difficulties occasioned by the exceptional circumstances of the infirmary, viz., the transitional state of the nursing arrangements, pending further development of the scheme for their reorganisation. They have also taken into consideration the fact that these difficulties were augmented by the injudicious conduct of the medical officer, on which the Board have already commented. But in some important instances the Board fail to find any sufficient justification of the matron's conduct. This is especially so in the case of Nurse Pinder, who was kept on continuous duty for a period of 24½ hours, and the Board consider the matron's conduct deserving of severe censure. It is obvious that the administration of the infirmary cannot be permitted to suffer, as it must have done in the past, in consequence of the relations existing between the medical officer and the matron, and the Board have had considerable doubt as to whether, under the circumstances, they would be justified in permitting either the medical officer or the matron to continue in office. They have, however, determined not to adopt the extreme course of requiring their resignations, but to allow them both to continue in office for a limited period, provided that they give a distinct undertaking that they will in future work amicably together in the interest of the institution, and on the clear understanding that if the friction continues one or both of them must cease to hold office. The Board will address a further communication to the Guardians in regard to the future administration of the infirmary.

The Board decided to hold a special meeting for the consideration of the letter next Tuesday, at which the medical officer and matron will be present.

### Death in Our Ranks.

We regret to announce the sudden and unexpected death of Miss Ada C. Swiney, the third daughter of the Rev. A. Swiney, Bradfield St. Clare Rectory, Suffolk, from heart failure after an attack of pneumonia, at the Leeds Union Infirmary, on October 9th. Miss Swiney had entered for the three years' course of training as a probationer, and completed her first year in May of this year.

### Wants and Workers.

A serviceable Bath chair is greatly needed for the use of the sick and infirm in a very poor parish. Anybody who has a secondhand one in good condition to give away is asked to write to the Rectory, St. Benedict's, Ardwick, Manchester; or to H. F. Gethen, 52, St. John's Wood Road, London.

### Burdett's Official Nursing Directory.

IMPORTANT TO NURSES.

In reply to inquiries, we have to state that the schedules of nurses who have not yet returned them will be in time for the first issue of "Burdett's Official Nursing Directory" if sent in at once to the offices (28 & 29, Southampton Street, Strand, W.C.). At the same time, we would point out that a nurse who has taken the trouble to revise the schedule sent her, and to bring the particulars up to date, will naturally appear as a more experienced woman than one who, despite every effort on the part of the Editorial Committee, has made it impossible to bring the information concerning her up to date. The "Directory" is now in a forward state of preparation and progress, and it will be published shortly. All nurses, therefore, who are wise, and who realise the importance to themselves of supplying the medical profession and the public with accurate and full particulars concerning their qualifications, wil without delay, we are confident, either call at the office and see the sub-editor or look up and return the schedules sent them.

### Everybody's Opinion.

[Correspondence on all subjects is invited, but we cannot in any way be responsible for the opinions expressed by our correspondents. No communication can be entertained if the name and address of the correspondent is not given, or unless one side of the paper only be written on.]

THREE HUNDRED ORPHANS IN CAGES.

Mr. Thomas F. Raven, medical officer to St. Mary's Orphanage, Broadstairs, writes: Under the amiable heading of "Orphans Caged" you insert in your issue of October 3rd a communication from a "Medical Correspondent." Your "Medical Correspondent" takes upon himself, as a good many other people do, the office of censor of the Kilburn Sisterhood. He poses as the protector of the "poor infants" from the cruelties that are practised upon them, and the dangers to which they are exposed by the mismanagement of the Sisters of the Church. His lofty tone would be deeply impressive if he did not distort facts, and if his animus had not led him to wilfully misrepresent the state of things at St. Mary's Orphanage at Broadstairs. His quarrel is with the wire cubicles that are in use at St. Mary's. He states that all these structures must be unfastened, singly, to allow of the escape of the children in the event of fire. This is not the fact; a large number of them open at once by a very simple mechanical contrivance. Those that do not open in this way can be rapidly unfastened by any of the Sisters. Your correspondent is at liberty to hold his own opinion on the question of danger from fire; on the other hand, the Sisters of the Church, even he may allow, are entitled to hold theirs. They have, perhaps, given the matter a little more attention than he has, and their opinion is that, in the event of fire, the control that they would be able to exercise over the children by restraining the effect of panic—such as a confused stampede—would be of much greater importance than the possible loss of a few minutes of time. That is their opinion. Possibly your correspondent may concede that they hold it honestly, and that they may have given the matter some consideration. I say that he wilfully misrepresents what he saw, or what he says he saw, at St. Mary's Orphanage. When he has seen in travelling menageries wild beasts' cages which contain excellent and comfortable beds and bedding, together with necessary utensils and conveniences for the toilet, and when he has seen such cages decorated with cards, pictures, and ornaments, then and not till then will he be justified in stating that the cubicles at St. Mary's bear a "strong resemblence," or indeed any resemblance at all, to the cages of a "travelling menagerie." He says that they are "euphemistically called cubicles." Where is the euphemism? They are perfectly airy, wholesome, comfortable partitions for sleep and rest. Let him consult a shilling dictionary. He indulges in some moral reflections at the close of his letter at the expense of the "good Sisters," as he benevolently calls them. "Is," he asks, "the substitution of mechanical restraint for loving personal care worthy of a community of Christian workers at the end of the nineteenth century?" To which I reply, loving personal care of poor children is the main object and work of the Sisters of the Church, and it is no more interfered with by the mechanical restraints upon the children which so greatly shock your correspondent than it would be by their being tucked up in bed. He asks this question. I will ask another. Is the garbling of facts and the anonymous misrepresentation of a body of women who devote their lives to the welfare of poor and destitute children worthy of a "Medical Correspondent" at the end or the beginning of the nineteenth or any other century?

"Nurse A. B." writes: As a hospital nurse who has worked for over a year in the spinal ward of St. Mary's Home, Broadstairs, I read at first with curiosity and then indignation a letter headed "Orphans Caged" in The Hospital of the 3rd inst. In this article "Medical Correspondent" sets forth the disadvantages of the cubicles used as sleeping compartments by the children at St. Mary's Home. I should like to mention some of their advantages. To the elder girls they afford a separate bed-room, so that they can rise, wash, and dress in privacy. "Medical Correspondent" states: "At Broadstairs each cubicle must be opened singly." This is incorrect; of the 300 cubicles in the Home about 30 open as he describes (being the cubicles in the infirmary and some in the babies' dormitory) to enable the Sisters to give individual attention without opening an unnecessary number of doors, but in the larger dormitories the simultaneous opening of the different rows of cubicles is adopted, and a Sister sleeps at hand in every case. The orphans' fire brigade is a practical one, the girls answering an unexpected call with a promptitude that quite satisfied the fire brigade superintendent. In case of fire in a large institution the danger of panic is often worse than the actual fire. Thanks to the cubicles, a rush would be impossible, and the staff of Sisters could release the children at their discretion. Fire is a possibility, pneumonia would be a certainty if the little ones between the ages of three and five were able to get out of bed as soon as it is daylight. If "Medical Correspondent" were an inmate of St. Mary's Home for a week, he would see that every child has a mother's care and would not suggest that more "loving personal care" was needed. If the orphans are "caged" at night, they suggest to a fair, impartial spectator, happy birds rather than animals in a menagerie.

[We are afraid "the opinion of our correspondent and of the Sisters of the Church" can matter but little to the poor children who, in case of fire, might be roasted alive in these shameful cages. The defenders of the Sisters of the Church who so love restraint—for others—have evidently but one scribe amongst them, if we may judge by the wording of the letters which reach us. It is matter for regret that any medical man can be found to defend this systematic caging of children when public and medical opinion are unanimously against practices of this kind which have long since disappeared from every well-administered institution.—Ed. *T. H.*]

### Minor Appointments.

Sheffield Union Infirmary.—Miss Edith M. Holt has been appointed Nurse-in-Charge of the maternity ward at this infirmary. Miss Holt was trained at the Derby Royal Infirmary, and has also worked at the Bristol Royal Infirmary as assistant nurse, and at the East End Mothers' Home, Stepney.

Royal Albert Edward Infirmary, Wigan.—Miss Frances Bruce Butchard and Miss Agnes McCaw have been appointed Sisters at this hospital. Miss Butchard was trained at the Royal Infirmary, Dundee, and has since been working in connection with the Torquay Nurses' Institute. Miss McCaw was trained at the Adelaide Hospital, Dublin, and has held the appointment of charge-nurse at the North-Eastern Fever Hospital, London.

### Appointments.

### MATRONS.

Tiverton Infirmary and Dispensary.—Miss Mary D. Stephenson has been appointed Matron at this hospital. Miss Stephenson received her training at Charing Cross Hospital, where she has since held the position of Sister-in-Charge.

Bridgend Hospital, Glamorganshire.—Miss Mary Barwick has been appointed Matron of this new hospital. She received her training at the General Infirmary, Leeds, worked as sister at the Salop Infirmary, and as night superintendent at the Royal Infirmary, Aberdeen, and has held the post of matron at the Kincarrathie Asylum, Perth. Miss Barwick takes many good wishes with her to her new work.

Kettering Isolation Hospital.—Miss Mildred Lanyon has been appointed Matron to this institution by the Kettering Urban Council. Miss Lanyon was trained at the London Homœopathic Hospital, and has worked on the staff of the Hanover Institute for Nurses. For the last few months she has been acting as matron of the High Wycombe Small-pox Hospital, now closed.

Children's Convalescent Home, Gilmerton, Midlothian.—Miss Shortrede has been appointed Matron of this home. She was trained at the Royal Hospital for Sick Children, Edinburgh, and afterwards gained a three years' certificate from the Victoria Infirmary, Glasgow. Miss Shortrede also held the post of Sister at the Victoria Infirmary for eighteen months, and for the past two years has acted as Assistant Matron to the New Royal Hospital for Sick Children, Edinburgh.

### Notes and Queries.

*Madeira.*

(21) Is there any hospital or nursing institute in Madeira where nurses could get employment in the winter season? Or at any Swiss resort? Can you tell me anything of the Hollond Institute at Paris and Nice?—*J.M.L.*

There is the Seamen's Hospital at Madeira with eight beds and one nurse besides the matron, Miss Bulens. We do not know of a nursing institute in Madeira, neither have we knowledge of any in Switzerland. Such institutions, where they do exist abroad, have no difficulty in finding nurses when they want them, and the chances of foreign appointments are small in proportion to the numbers of nurses who wish to "go abroad." The managers of the Hollond Institute are now advertising in our columns for nurses. See last week's "Nursing Mirror."

*Training.*

(22) Can you tell me of any children's hospitals or convalescent homes where probationers are taken at the age of 18 years? No premium.—*Miss M. H.*

You will find a list of hospitals and homes, with the ages at which probationers are received, in "How to Become a Nurse" (Scientific Press, 28 & 29, Southampton Street, Strand, London, W.C.) Write to Miss Lobb, Uplands, Loughton, Essex, who trains young probationers at her children's home.

*Nursing Abroad.*

(23) I wish to obtain a post abroad. What steps should I take?—*Nil Desperandum.*

Why do you not read the replies we constantly give to the question you ask? Watch the advertisements in our columns or advertise yourself. You will find the names of foreign hospitals and institutions given in Burdett's "Hospitals and Charities," and we can only suggest your writing to the matrons for information as to possible openings.

*Colonial Nursing Association.*

(24) Can you tell me anything about the Colonial Nursing Association? I should be glad to know the proper quarters to make application, as I have not seen any news about it in the nursing papers.—*M. M. H.*

We are afraid you do not read your Hospital. If you will refer to the "Nursing Mirror" for July 18th last you will find an account of the Colonial Nursing Association. Mrs. Piggott is the hon. secretary, and the address is Imperial Institute, S.W., but we have been asked by Mrs. Piggott to say that she is overwhelmed with applications from nurses, receiving many more than it is possible to answer at present.

*Nursing in Shanghai.*

(25) Are any more nurses wanted for Shanghai? If so, to whom should I apply?—*Trained Nurse.*

Many nurses are asking this question. See reply to similar query in this column last week.

*Fever Hospitals in France.*

(26) Can you tell me the addresses of any Fever Hospitals in France, Normandy if possible? Would English nurses be able to obtain employment at them?—*S. D. and C. B. A.*

You had better write to the Central Administration Offices of the *Assistance Publique*, the French Hospital Authority, 3, Avenue Victoria, Paris.

*Professor Michael Foster's Address.*

(27) Can you tell me whether the lecture given by Professor Michael Foster at Charing Cross will appear in any medical papers?—*Aidyl.*

You will find an abstract in the *British Medical Journal* and *Lancet* of last week.

